# Genome plasticity is governed by double strand break DNA repair in Streptomyces

**DOI:** 10.1038/s41598-018-23622-w

**Published:** 2018-03-27

**Authors:** Grégory Hoff, Claire Bertrand, Emilie Piotrowski, Annabelle Thibessard, Pierre Leblond

**Affiliations:** 1 Université de Lorraine, Institut National de la Recherche Agronomique, Dynamique des Génomes et Adaptation Microbienne (DynAMic), UMR INRA 1128, Nancy, 54000 France; 2 Present Address: Microbial Processes Interactions (MiPI), Gembloux Agro-Bio Tech, Bât. G1 Bio-industries, Passage des Déportés, 25030 Gembloux, Belgium

## Abstract

The linear chromosome of the bacterium *Streptomyces* exhibits a remarkable genetic organization with grossly a central conserved region flanked by variable chromosomal arms. The terminal diversity co-locates with an intense DNA plasticity including the occurrence of large deletions associated to circularization and chromosomal arm exchange. These observations prompted us to assess the role of double strand break (DSB) repair in chromosome plasticity following. For that purpose, DSBs were induced along the chromosome using the meganuclease I-*Sce*I. DSB repair in the central region of the chromosome was mutagenic at the healing site but kept intact the whole genome structure. In contrast, DSB repair in the chromosomal arms was mostly associated to the loss of the targeted chromosomal arm and extensive deletions beyond the cleavage sites. While homologous recombination occurring between copies of DNA sequences accounted for the most part of the chromosome rescue events, Non Homologous End Joining was involved in mutagenic repair as well as in huge genome rearrangements (i.e. circularization). Further, NHEJ repair was concomitant with the integration of genetic material at the healing site. We postulate that DSB repair drives genome plasticity and evolution in *Streptomyces* and that NHEJ may foster horizontal transfer in the environment.

## Introduction

The soil bacterium *Streptomyces* is renowned for its biotechnological capabilities, most notably its ability to synthesize a wide variety of bioactive secondary metabolites used in medicine as antiproliferative agents (antibiotics, anticancer and antifungal drugs)^1^. Furthermore, this group of bacteria possesses unique genome characteristics among which a high GC content (circa 72%) and a large linear chromosome (6–12 Mb) ended by inverted repeats. Besides, the chromosome is highly compartmentalized, with a conserved central region flanked by variable arms^2,3^. The areas located between the conserved and the specific regions show a progressive loss of synteny ensuing from an accumulation of insertion/deletion events^4^. One very intriguing phenomenon which has been known for decades is the high genetic instability in this genus. Thus, during culture, *Streptomyces* species offsprings present between 0.1 and 1% of spontaneous mutants defective in morphological and physiological differentiation including secondary metabolic pathways^5^. This phenotypic instability correlates with the formation of large deletions affecting the chromosomal arms. Deletions have been found to be associated with chromosomal circularization^6,7^, chromosomal arm exchange^8,9^ and fusion of chromosomal ends^10^. High copy number DNA amplification was also associated with the formation of deletions^11–13^. Moreover, homologous recombination (HR) and illegitimate recombination (IR) are known to be involved in such chromosome rearrangements^8,12^. These recombination events could also bring into play plasmid/chromosome exchanges resulting in hybrid chromosome formation^14,15^.

Most of the key actors of HR in many model eubacteria (*e.g*. RecA) have orthologues in *Streptomyces*^16,17^. Yet, there are some striking discrepancies; for example, the presence of AdnAB as the main helicase nuclease activity instead of RecBCD or the presence of alternative post-synaptic actors to RuvABC and RecG^18,19^. However, HR is effective in *Streptomyces*, and as in many other bacteria, is assumed to contribute to genome stability as an error free repair mechanism^20^. On the other hand, HR can occasionally lead to major chromosome structure alteration when occurring between ectopic homologous copies^8,9^.

Non Homologous End Joining (NHEJ), as an IR mechanism, does not need any intact template to resolve Double-Strand Break (DSB) repair and can directly ligate the broken ends together after a facultative end processing step. This repair mechanism can lead to major chromosome rearrangements and is potentially mutagenic at the recombination site^21^.

The NHEJ pathway was long considered as the hallmark of eukaryotes, suggesting that DSB repair in prokaryotes fully relies on HR. However, early bioinformatics studies led to the identification of orthologues of a key NHEJ actor (*i.e*. Ku proteins) in several phylogenetically distant bacterial genomes^22,23^. While the NHEJ pathway was deciphered in model bacteria such as *B. subtilis* or *M. tuberculosis*^24,25^, we have recently shown that *Streptomyces* possess a large set of NHEJ-like genes with some of these being conserved (the ‘core’ NHEJ gene set) between species and strains^26^. Surprisingly, the involvement of all NHEJ-like genes, including the ‘variable’ set, in DNA damage response in *Streptomyces* suggests that in these organisms, a more complex pathway than in *Mycobacterium* or *Bacillus* is present.

Here we aimed to assess the role of DSB repair mechanisms (HR and NHEJ) in the formation of large DNA rearrangements and in chromosomal structure evolution in *Streptomyces*. To achieve this, we induced the formation of chromosomal DSBs using an I-*Sce*I mediated system and surveyed the chromosomal structure of the survivals.

## Results

### DSB repair in the central part of the chromosome is mutagenic

**Figure 1:**
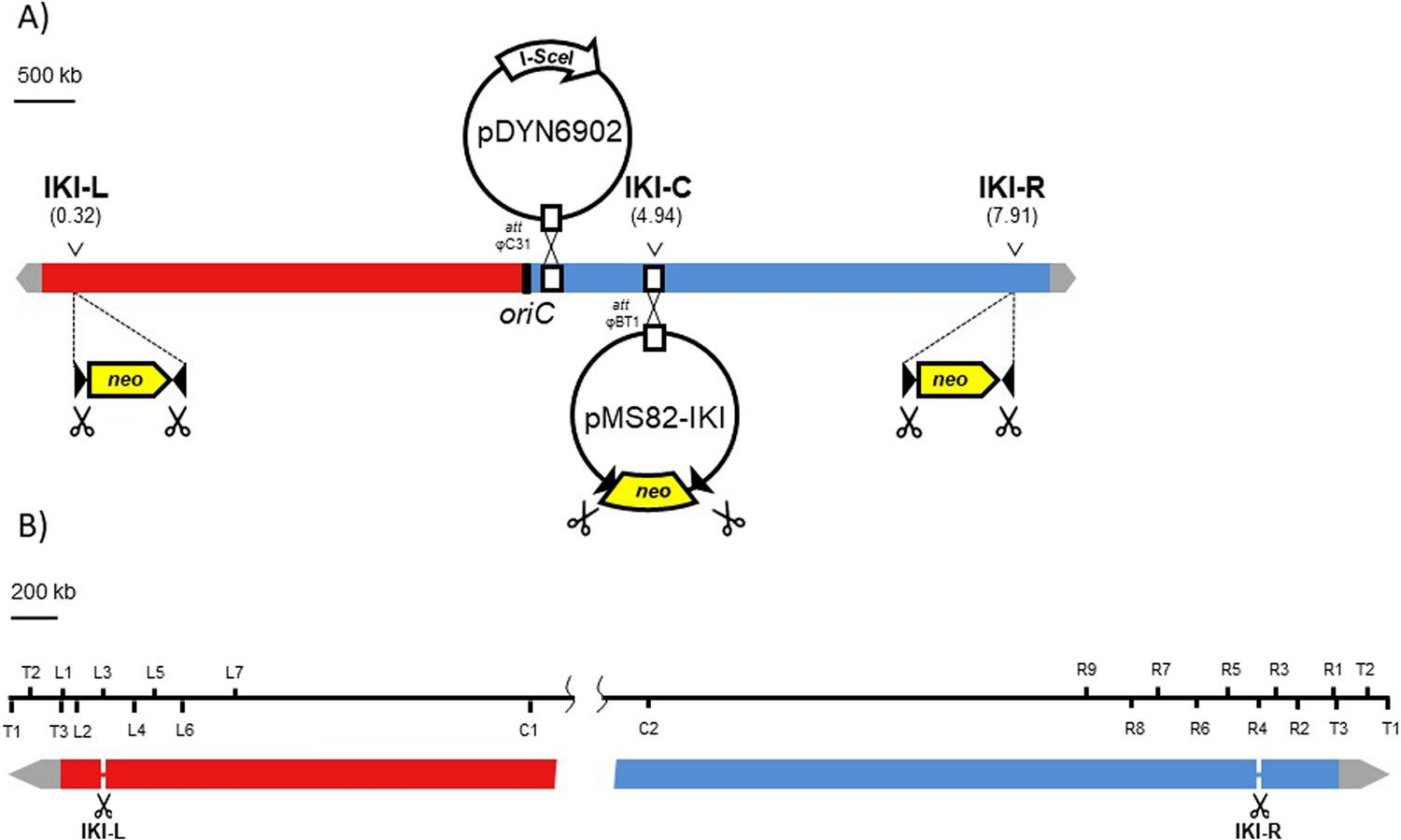
I-*Sce*I DSB induction and chromosomal survey device. (**A**) I-*Sce*I mediated DSB targeting in *S. ambofaciens* chromosome. The target sequences for I-*Sce*I meganuclease are carried by the IKI cassette designed with *neo* gene, conferring resistance to kanamycine, flanked by two convergent I-*Sce*I restriction sites (dark triangle). Red and blue boxes represent left and right replichores of the chromosome. Grey arrows represent the terminal inverted repeats at each chromosome extremity. The IKI cassette was integrated either in the core genome (IKI-C, position 4.94 Mb), in the left chromosomal arm (IKI-L, position 0.32 Mb) or in the right chromosomal arm (IKI-R, position 7.91 Mb). The cassette was inserted at IKI-L or IKI-R loci through homologous recombination. The IKI-C locus corresponds to the attachment site of phage φBT1 (attB φBT1) where pMS82 conjugative plasmid carrying IKI cassette (therefore named pMS82-IKI) was integrated. Meganuclease encoding I*-Sce*I gene was carried by pDYN6902 conjugative plasmid and integrated in the chromosome at the attachment site of phage φC31 (attB φC31). Scissors symbolize the DSB (I-*Sce*I restriction sites) triggered by the meganuclease. (**B**) Distribution of the loci used for chromosome mapping after DSB induction. T stands for loci included in the TIRs. L, C and R for left, central and right loci, respectively. The data analysis is schematically represented on Fig. 4.

To monitor the consequences of DSB repair along the *Streptomyces* chromosome, we developed a DSB inducible system based on the meganuclease I-*Sce*I heterologous expression (see Methods). A target cassette named IKI consisting of the *neo* gene, which confers resistance to kanamycin, flanked by two I-*Sce*I sites, was introduced in the central region of the chromosome (IKI-C locus, position 4.94 Mb) of *Streptomyces ambofaciens* ATCC 23877 (Fig. 1). I-*Sce*I endonuclease was constitutively expressed from an integrating plasmid in the chromosome. The functional activity of the endonuclease was proved by the loss of the target IKI cassette or the I-*Sce*I gene in at least 79% of the progeny when selective pressure was lifted (Table S1).

**Figure 2:**
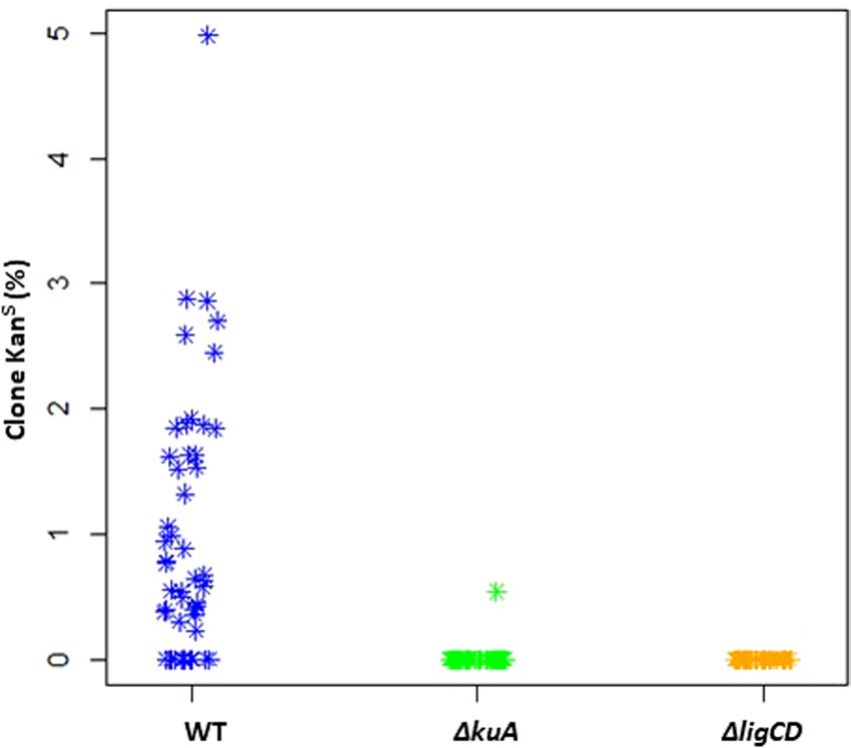
Kanamycin sensitive (Kan^S^) clones appearance after DSB generation at IKI-C. Proportion of Kan^S^ clones obtained in each independent lineage (symbolized by a flake) of WT, *∆kuA* and *∆ligCD* contexts. For each of the 50 lineages, an average of 230 clones was analyzed by replica-plating with and without kanamycin.

**Figure 3:**
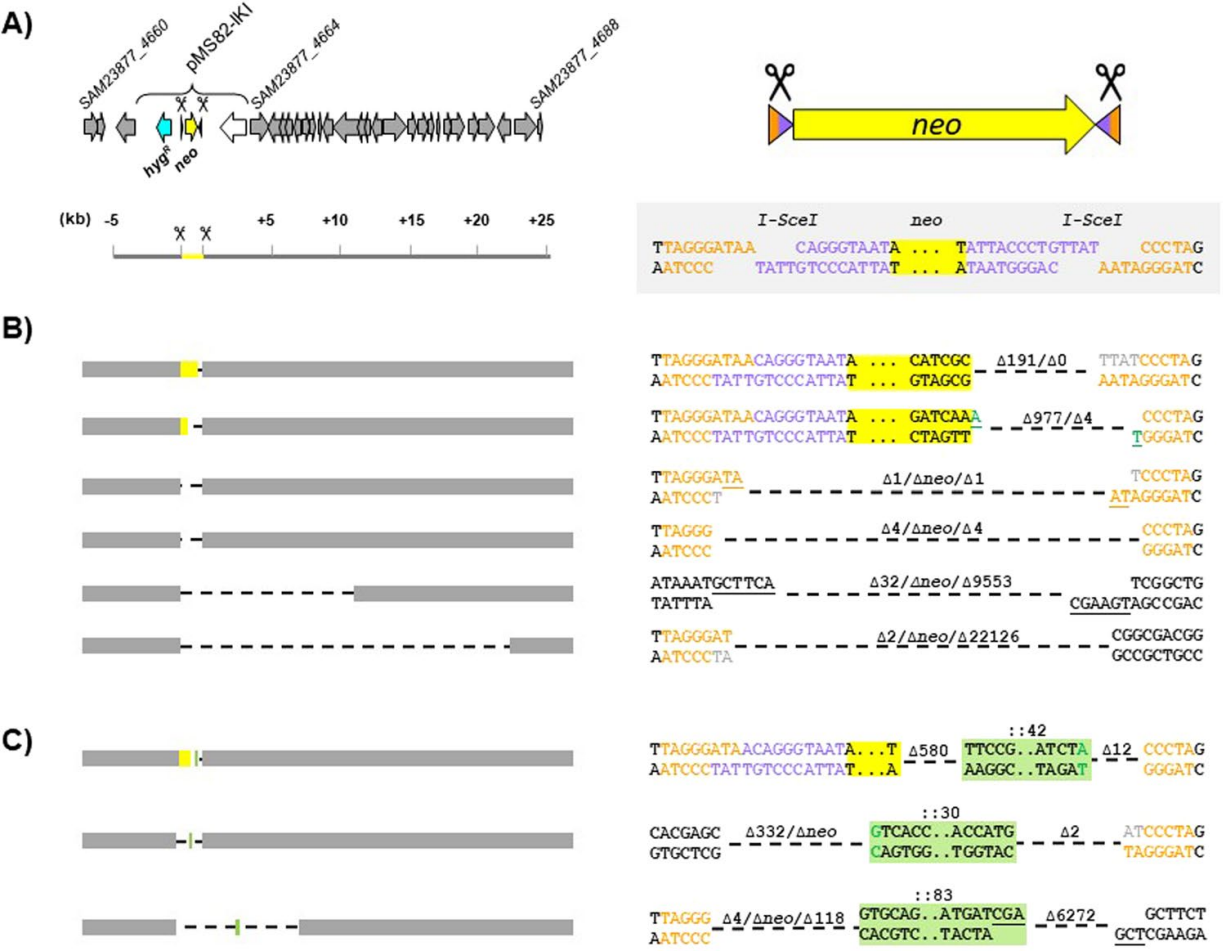
DNA repair features after DSB generation at IKI-C. (**A**) IKI-C locus: the left panel shows the 30 kb region surrounding the insertion site of pMS82-IKI at IKI-C locus. Scale is in kilobases. Locus_tags of the CDS delimiting the region are indicated according to Thibessard *et al*.^31^. The right panel focuses on the IKI cassette structure and sequence. Since the I-*Sce*I recognition site is a non palindromic sequence, the resulting restricted ends are distinguished by two different colors (orange and purple). The *neo* gene is colored in yellow while hygromycin resistance gene (*hyg*^R^) is colored in cyan. All DNA repair event examples are represented at these two different scales (see B). (**B**) Examples of deletion events occurring at IKI-C at the kilobase (left panel) and nucleotide (right panel) scales. Grey boxes represent the region flanking IKI-C cassette. Deleted regions are symbolized by dotted lines. The ∆ symbol indicates the number of deleted nucleotides at each restricted end. When the ∆ symbol is followed by *neo*, it indicates a complete deletion of the kanamycin resistance gene. (**C**) DNA integration cases. Nucleotides added by fill-in synthesis are colored in grey. Nucleotides added by non-template addition are colored in green. DNA fragments integrated during repair are boxed in green. Microhomologies present at the repair scar are underlined.

We surveyed the fate of DSBs in the central region by assessing the occurrence of Kan^S^ clones which were expected to result from the unfaithful repair of I-*Sce*I cuts at one or both sites of the IKI cassette. To test this hypothesis, fifty independent wild-type lineages harbouring the IKI cassette and expressing I-*Sce*I were selected and their progeny was tested for kanamycin sensitivity (Fig. 2). An overall average of 1.1% of Kan^S^ clones was observed (82 Kan^S^ clones out 7,420 colonies); each lineage produced between 0 and 10 Kan^S^ colonies. The spontaneous Kan^S^ frequency in a strain carrying the IKI locus but not expressing I-*Sce*I was significantly lower (<0.02%; 0 out of 5,768 colonies tested, P < 0.01). In order to characterize the repair features, we mapped the IKI locus in each Kan^S^ clone by amplifying the whole region and determining the nucleotide sequence of the scar. In all but two cases, the amplification patterns differed from the WT reference and dramatically varied in size revealing deletions estimated to expand between 191 bp and 23,591 bp. In the last two Kan^S^ clones, no amplification pattern could be obtained indicating that the deletion was even larger. Given the selective pressure applied to maintain the IKI cassette carrying vector (hygromycin resistance), deletions could not extend beyond the *hyg*^*R*^ gene located about 500 bp upstream the 5′ I-*Sce*I site (Fig. 3A). Thus, downstream the 3′ I-*Sce*I site, the deletion size reflected the efficiency of the nuclease activity and revealed the dispensability (under laboratory conditions) of the genes included in that chromosomal region (Fig. 3B). Scar sequencing showed that repair events modified one or both I-*Sce*I sites, except for two clones in which both I-*Sce*I sites remained intact despite the deletion of the *neo* gene. Repair appears to be mediated by micro-homologies of between 1 and 6 nucleotides in 50% of the scars. A nucleotide addition was observed in 40% of the scars and corresponded to a single non-templated nucleotide addition on blunt ends or to a fill-in synthesis of 1 to 4 nucleotides at the 5′ recessed ends. In summary, the scars displayed signatures of a classical NHEJ, as observed in *Mycobacterium tuberculosis*^27^. This study provides the first argument in favor of a functional NHEJ repair pathway in *Streptomyces*.

In three Kan^S^ clones, the scar analysis revealed the presence of a DNA insertion at the repair point (Fig. 3C). In two cases, the inserted sequence (42 or 83 bp) was originating from a region close to IKI-C. In the third case, the 30 bp sequence perfectly matched to the 5′ end of a transposase gene located in the terminal inverted repeats (TIR).

### Mutagenic DSB repair in the central part of the chromosome relies on the NHEJ pathway

In order to confirm the involvement of the NHEJ pathway in DSB repair, we transferred the I-*Sce*I device (enzyme and target cassettes) into strains that were defective for the bacterial NHEJ machinery (deficient for the production of KuA, the main Ku protein, or the ATP-dependant ligases LigC/LigD proteins). Hence, we previously showed that these NHEJ-like genes were involved in the response to genotoxic stress^26^. As for the WT background, fifty independent lineages per mutant were isolated and screened for Kan^S^ clones. In contrast to the WT, only one Kan^S^ clone was obtained from *∆kuA* lineages and no Kan^S^ clone was detected in the *∆ligCD* context (Fig. 2) which was significantly different from the WT (*t* test, α < 0.05). These data confirmed the key role of KuA at the expense of KuB and KuC although both were involved in the cellular response to DNA damaging agents^26^. It also confirmed that the ligases C and/or D are involved in the illegitimate DSB repair. The unique Kan^S^ clone isolated from the *∆kuA* background revealed the existence of an alternative illegitimate recombination pathway potentially involving the other two Ku proteins (KuB or KuC). Hence, alternative end joining pathways have been reported in *E. coli* which is devoid of the canonical NHEJ actors Ku and ATP-dependent DNA ligase LigD^28^, as well as in eukaryotes (from yeast to human,^29,30^.

### DSB repair in the chromosomal arms generates genome wide rearrangements

**Figure 4:**
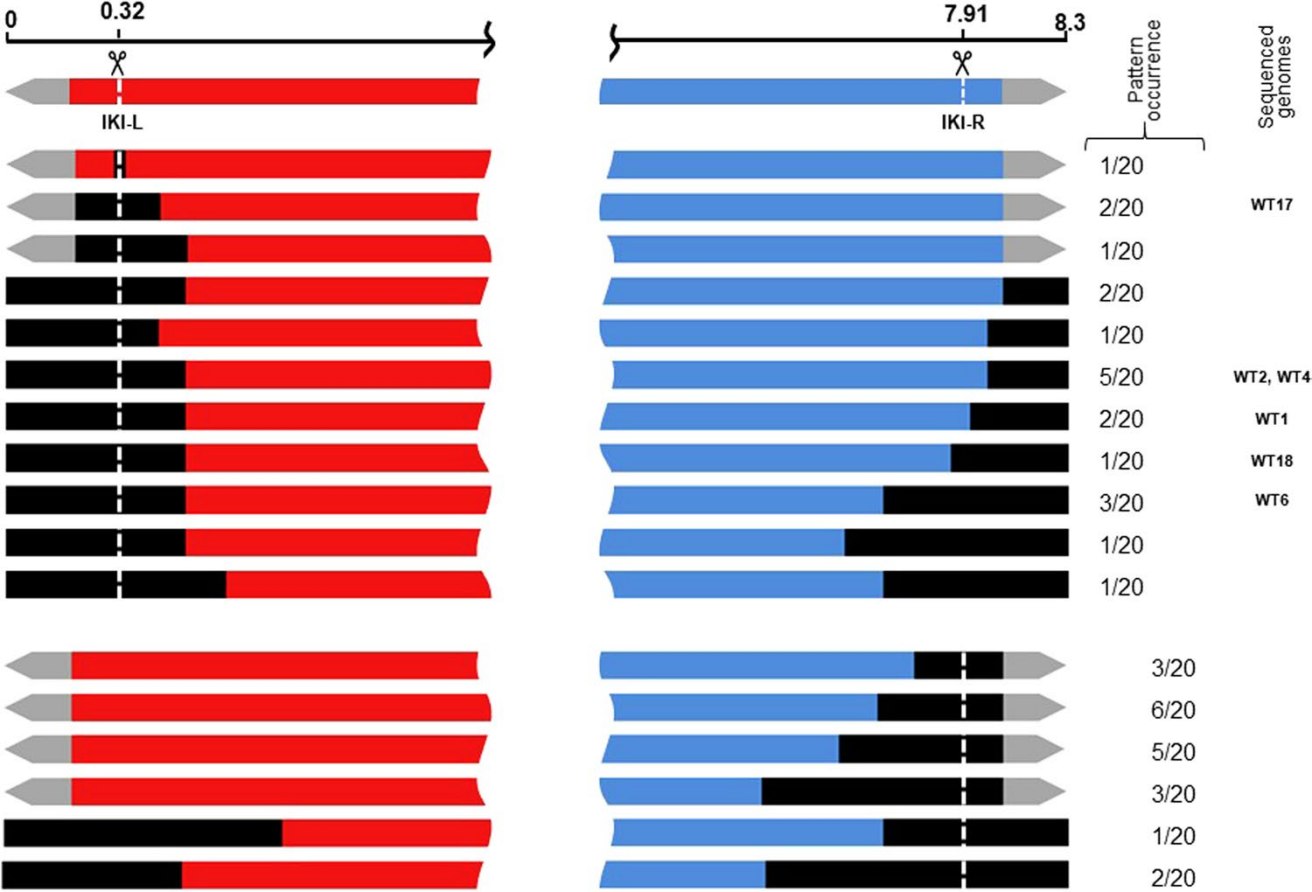
DNA rearrangements occurring in survivors after DSB induction at IKI-L and IKI-R. The chromosome of survivors were mapped using a set of targets for PCR amplication distributed along the chromosome (Fig. 1B). The scale is given in Mb. Grey arrows represent the terminal inverted repeats at each chromosome extremity. The DSB sites (IKI-L or IKI-R) are represented by the vertical white dotted lines. The black boxes represent the deleted areas. The occurrence of each deletion pattern (among 20 strains analyzed per chromosomal arm) and the strains chosen for whole genome sequencing are mentioned on the right.

DSBs were generated at two sites in the arms of the WT chromosome: IKI-L (position 0.32 Mb) and IKI-R (position 7.91 Mb; Fig. 1). Twenty lineages were subcloned for each insertion site. Contrasting with DSBs induced in the central part of the chromosome, more than half of the lineages displayed heterogeneous colonial phenotypes at this stage (Fig. S1) and needed further subcloning (up to eight) to reach morphological homogeneity. Once stabilized, the 40 lineages exhibited a wide range of morphologies (Fig. S1C). All but one were Kan^S^. Their chromosomal structures were analyzed through PCR amplification of loci distributed along the chromosome (Fig. 1B). While the loci observed in the centre of the chromosome were systematically present, several loci were simultaneously absent in one or both chromosomal arms suggesting the occurrence of large deletions (Fig. 4). Since chromosome circularization events have frequently been reported in *Streptomyces*^5^, deletions affecting both ends were considered to be indicative of such events. Interestingly, circularization appeared more frequent after DSB in the left arm (16 out of 19) than in the right one (3 out of 20, exact Fisher test p < 0.01). As a control, the genome stability of 30 WT lineages carrying the I-*Sce*I gene but not the IKI-cassette was assayed: two out of the 30 strains showed a terminal rearrangement, which is consistent with the previously characterized instability level in *S. ambofaciens*^5^. The same procedure (subcloning of 20 lineages and PCR analysis) was applied in the *∆kuA* and *∆ligCD* contexts after DSB induction at IKI-L. Circularization was indicated in 11 and 15 out of the 20 lineages derived from *∆kuA* and *∆ligCD* contexts, respectively. At the chromosomal structure level, no significant differences could be observed between WT and NHEJ deficient backgrounds.

**Figure 5:**
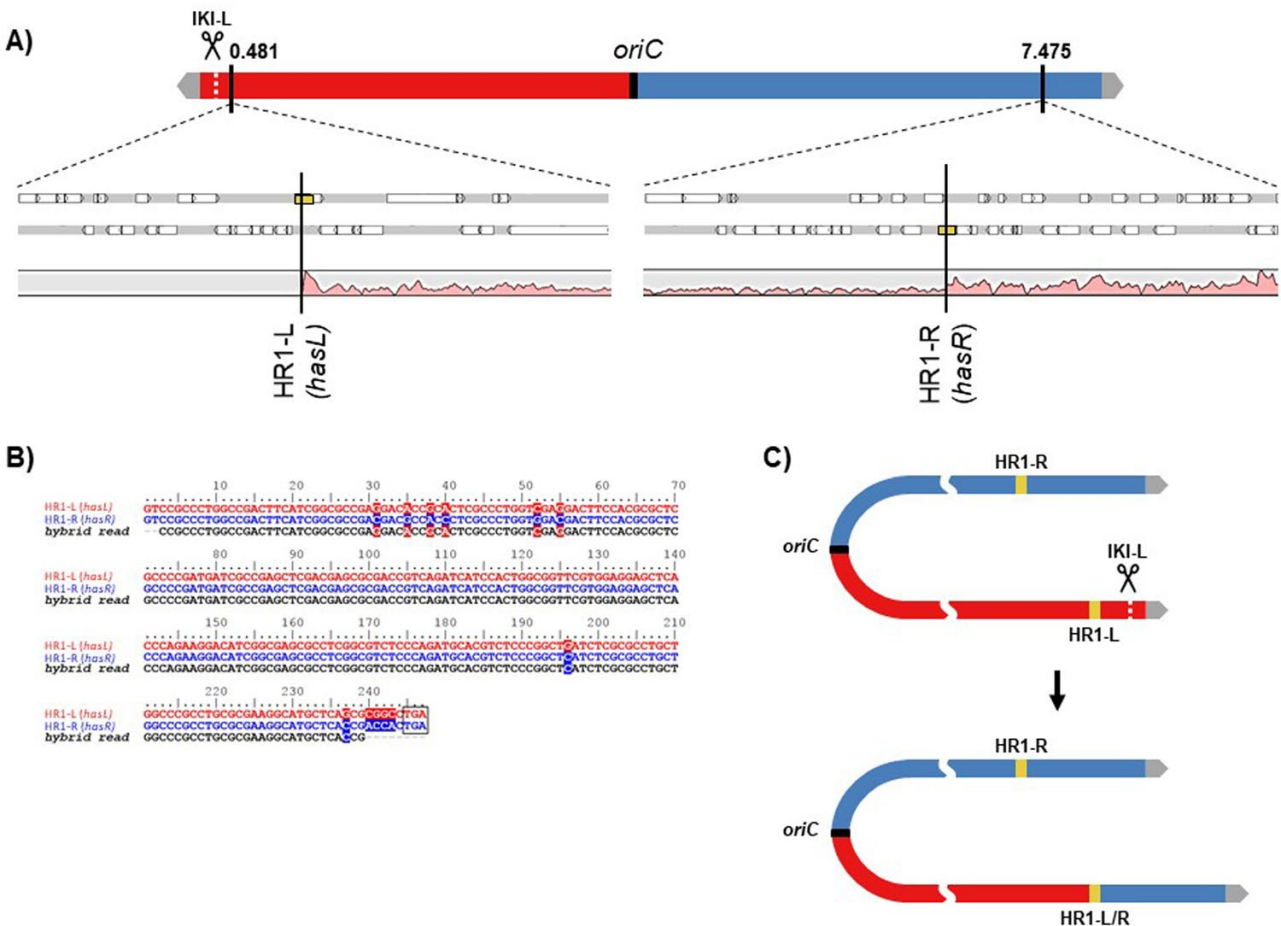
Chromosomal arm replacement by homologous recombination after DSB induction at IKI-L. (**A**) Analysis of the coverage of sequencing reads along the chromosome of a DSB survivor at IKI-L (WT17). Genome sequencing reads of strain WT17 plotted along the *S. ambofaciens* ATCC 23877 genome as a reference are shown as a pink area. Yellow boxes symbolize HR1-R and HR1-L (two duplicated copies of a sigma factor encoding gene: *hasL*, SAM23877_0517, and *hasR*, SAM23877_6930). (**B**) Hybrid read resulting from recombination between HR1-L and HR1-R sequences. HR1-L and HR1-R sequences were aligned with one typical hybrid_read found in WT17 sequencing data. The alignment shown covers the 3’ end of the *hasL/R* sequences (from position 600 to 846). Polymorphism is symbolized with a colored background. The stop codon is boxed. (**C**) WT17 chromosome structure. The hybrid HR1 sequence is labelled HR1-L/R. The I-*Sce*I cut at IKI-L resulted in the loss of the left chromosomal arm and in a homologous recombination event between HR1-R and HR1-L leading to the duplication of the right chromosomal arm over 825 kb.

Six lineages from each of the three genetic backgrounds (WT, *∆kuA*, *∆ligCD*) cut at IKI-L were chosen according to their diverse PCR patterns for whole genome sequencing (for sequencing data, see Table S2). Sequence reads were aligned on the chromosomal sequence of *S. ambofaciens* ATCC 23877 as a ref.^31^. This alignment confirmed that some areas of the chromosome were deleted (*i.e*. had no read coverage) while other showed an increased coverage level compared to the genome average (by a factor two or more) as illustrated in Fig. 5A. The extent of the deletions can reach several hundreds of kilobases (up to 2.1 Mb) contrasting with that observed in the central part of the chromosome. That could reflect (i) the absence of essential genes in the arms in laboratory growth conditions and (ii) the involvement of a highly efficient exonuclease activity.

**Figure 6:**
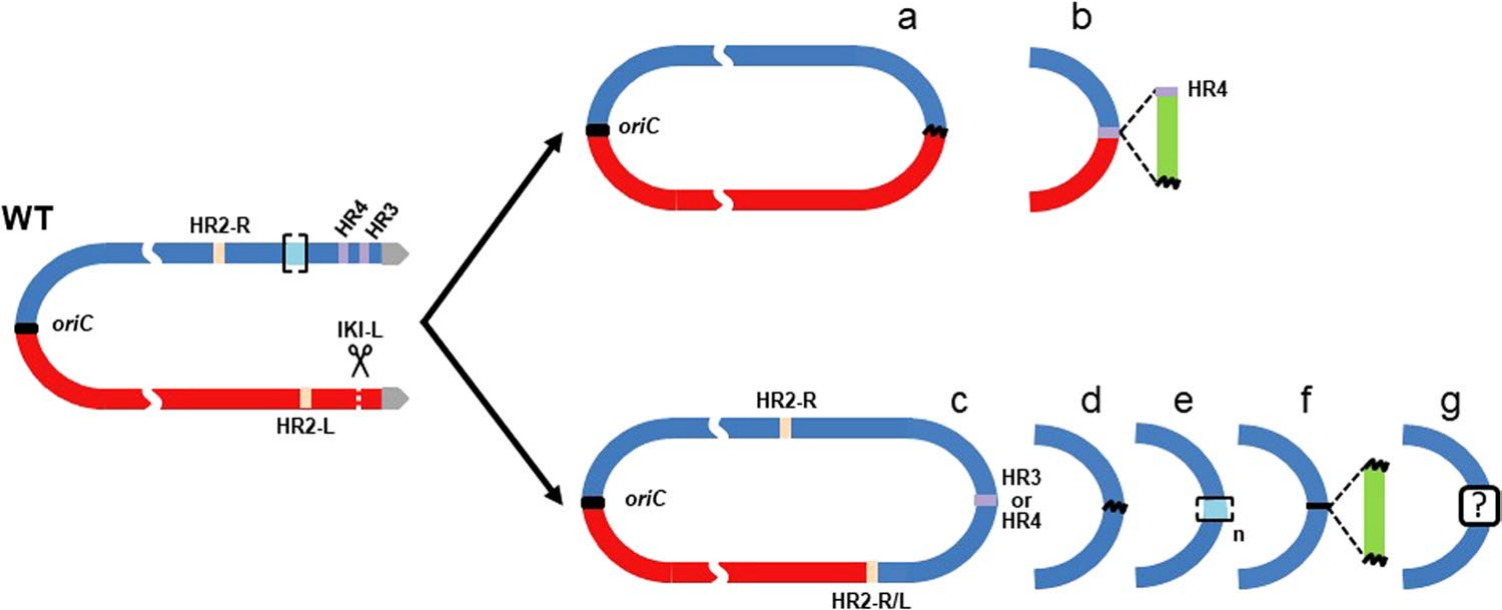
Chromosomal circularization after DSB induction at IKI-L. HR2-L and HR2-R are two homologous regions sharing over 99% nucleotide identity along 1,672 bp and are symbolized as salmon pink boxes. The hybrid HR2 sequence is labelled HR2-L/R. HR3 is a region including two identical transposase encoding genes (SAM23877_7417 and SAM23877_7422) in opposite direction. Similarly, HR4 is a region including two identical transposase encoding genes (SAM23877_7326 and SAM23877_7404) in opposite direction. HR3 and HR4 are symbolized as purple boxes. Illegitimate recombination scars are represented by black wave lines. Green boxes represent inserted DNA stretches. Amplified sequences are marked out by light blue boxes between brackets. In some cases, circularization occurred by simple event of fusion between sequences present on both arms with (**a**) or without (**b**) DNA insertion. The other cases are typified by a common feature, which consists in the deletion of the left arm until the HR2-L locus and its recombination with its homologous region on the right arm (HR2-R), leading to the formation of a hybrid HR2-R/L sequence. This arm replacement is concomitant with circularization through different molecular event: HR involving HR3 or HR4 (**c**), IR (**d**) sometimes accompanied by DNA amplification (**e**) or DNA insertion (**f**). For case e, the read coverage level of the amplified region prevents the identification of the fusion sequence and thus the nature of the recombination event. For case (**g**), the circularization may have occurred through recombination between two short homologous sequences (41 bp) in opposite direction.

In strain WT17 (Fig. 5A) the deleted area extended from the left chromosomal end to the *hasL* locus (deletion reaching 481 kb) while the area downstream *hasR* in the right arm had a two-fold coverage reflecting its duplication. Since *hasL* and *hasR* share 99% of nucleotide identity over their total length (846 nt,^8^), we called them HR1-L and HR1-R and suspected a HR event between them, leading to the replacement of the left arm by the right one. The existence of hybrid reads confirmed this hypothesis (Fig. 5B). The genome rearrangement led to the formation of new TIRs of about 829 kb *vs*. 210 kb in the reference strain (Fig. 5C). This repair event seemed to be the only one that maintained the linear configuration of the chromosome (4 out of 4 linear chromosomes, Table 1). The 14 other sequenced strains harboured a deletion at both chromosomal ends resulting in a circular configuration (Fig. 6). In some cases, circularization occurred by a simple event of fusion between sequences present on the two arms with or without DNA insertion at the fusion point (Fig. 6a,b). Both homologous and illegitimate recombination mechanisms were involved in these fusion events (Table S3). The other cases are typified by a common feature, which consists in the deletion of the left arm until a locus named HR2-L and its recombination with another locus named HR2-R, leading to the formation of a hybrid HR2-R/L sequence. HR2-L and HR2-R correspond to two copies (>99% nucleotide identity) of a 1,672 bp locus carrying two genes (SAM23877_6676 and SAM23877_6677) encoding a sigma factor and a hypothetical protein. The arm replacement resulted in the duplication of the region downstream the recombination point, similarly to the situation described in Fig. 5. However, in these latter cases, chromosomes were invariably circularized. Circularization occurred between sequences belonging to the two copies of the right arm *via* different molecular events (Fig. 6c,g) involving homologous and/or illegitimate recombination sometimes accompanied by DNA amplification or DNA insertion (Table S3). DNA amplification consisted of several hundred iterations of an amplifiable unit of DNA (AUD). These AUDs ranged from 14.2 to 32.8 kb in size and overlapped the stambomycin biosynthetic gene cluster (from SAM23877_7104 to SAM23877_7128).

**Table 1:** Genome features of survival strains after DSB induction at IKI-L in the WT and NHEJ^-^ contexts.

Strain	Conformation^a^	Deletion (kb)	Duplication (kb)	Insertion (kb)	Amplification^b^			Predicted genome size (Mb)^c^
					Size of the AUD (kb)	Estimated copy number	Estimated total size (kb)	
WT1	Circular (6 f)	876	767	9.7			—	8.20
WT2	Circular (6c)	785	864	—			—	8.38
WT4	Circular (6d)	827	761	—			—	8.23
WT6	Circular (6e)	1,140	498	—	14.7	590	8,7	~16.40
WT17	Linear	481	829	—			—	8.65
WT18	Circular (6d)	940	701	—			—	8.06
*∆kuA*1	Circular (6 g)	984	665	—			—	7.98
*∆kuA*6	Circular (6a)	683	—	—			—	7.62
*∆kuA*12	Circular (6e)	1,140	498	—	14.7	840	12,3	~20.00
*∆kuA*13	Linear	481	829	—			—	8.65
*∆kuA*14	Circular (6c)	800	783	—			—	8.28
*∆kuA*17	Linear	481	829	—			—	8.65
*∆ligCD*6	Circular (6b)	1,297	—	18.8			—	7.02
*∆ligCD*7	Circular (6e)	1,097	522	—	32.8	325	10,7	~18.40
*∆ligCD*9	Circular (6e)	1,140	498	—	14.7	820	12,1	~20.00
*∆ligCD*11	Linear	481	829	—			—	8.65
*∆ligCD*16	Circular (6c)	785	864	—			—	8.38
*∆ligCD*19	Circular (6a)	2,116	—	—			—	6.18

^a^The chromosomal conformation referring to Fig. 6 is indicated into brackets.

^b^AUD stands for Amplifiable unit of DNA. The copy number of the amplification was estimated considering the average coverage of sequencing reads of the amplified region relative to that of the whole genome.

^c^The genome size of the mutants carrying an amplification of DNA cannot be precisely evaluated.

**Figure 7:**
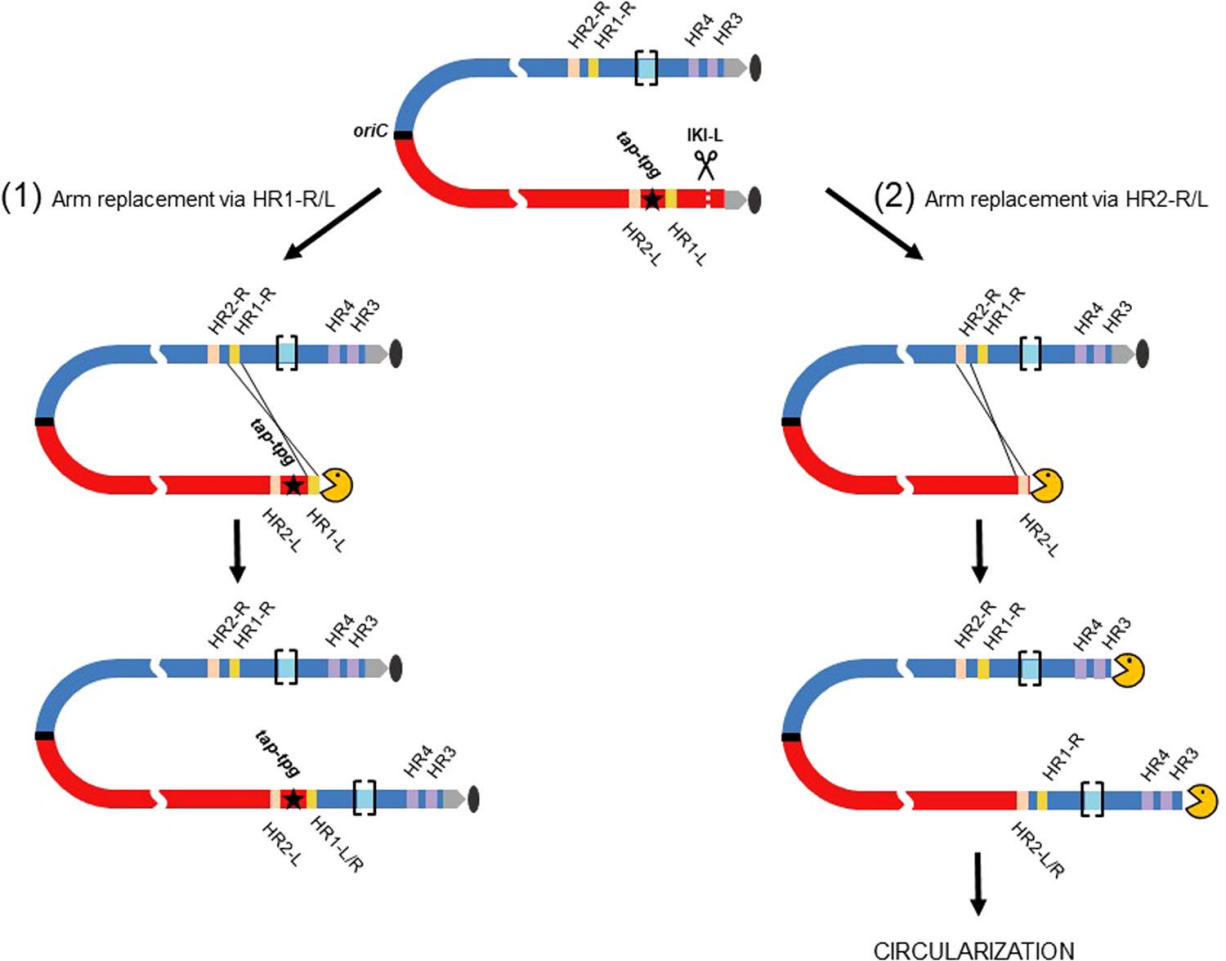
Fates of the chromosome structure following DSB and arm replacement. Red and blue boxes represent left and right replichores of the chromosome respectively. Grey arrows and the grey oval represent the terminal inverted repeats and the terminal proteins attached to them. HR2-L and HR2-R are symbolized as salmon pink boxes. The hybrid HR1 and HR2 sequences are labelled HR1-L/R and HR2-L/R respectively. HR3 and HR4 are symbolized as purple boxes. The AUD90 locus, susceptible to DNA amplification is marked out by light blue boxes between brackets. The *tap-tpg* locus encoding the terminal associated protein and the terminal protein respectively is represented by a black star. The yellow PacMan symbolizes the accessibility of the chromosome end to nuclease activities. After DSB induction at IKI-L, DNA resection progresses towards the inner parts of the chromosome concomitantly with the loss of the distal part of the arm. In case (**1**), DNA resection does not reach the *tap-tpg* locus, and arm replacement occurs by HR between HR1-R and HR1-L. Since *tap-tpg* locus is preserved, the linear configuration of the rearranged chromosome is stable. In case (**2**), DNA resection gets past *tap-tpg* locus and arm replacement is initiated by HR between HR2-R and HR2-L. The *tap-tpg* being deleted, this chromosome is unstable and irremediably leads to stabilization through circularization by different ways involving either HR3, or HR4 or an amplified sequence as previously shown.

A striking difference distinguished chromosome salvage events involving HR1 loci from those involving HR2 loci. While all the repaired chromosomes recombined through HR1 loci remained linear (4 out of 4), all the events involving HR2 loci became circular (11 out of 11). The location of genes encoding the terminal proteins Tpg and Tap involved in telomere maintenance (SAM23877_0536/0537) between both HR1 and HR2 recombination sites provides the most probable explanation for these contrasting situations (Fig. 7). Hence, arm replacement involving the HR2 loci remove the *tap*/*tpg* genes from the chromosome; in consequence, linearity can no longer be maintained and the only way out is circularization. In contrast, when arm replacement involved the HR1 loci, the *tap/tpg* locus remained present and chromosomal linearity was kept. This is consistent with the report of the circularization of *Streptomyces rochei* chromosome triggered by the loss of linear plasmids encoding the Tap-Tpg functions^32^.

Overall, these rearrangements led to genome size ranging from 6.18 Mb up to *c*. 20 Mb (reached in case of DNA amplification, Table 1).

## Discussion

Deciphering the mechanisms of genome plasticity in *Streptomyces* is pivotal to understand the biology of this soil bacterium in its competitive ecosystem and to exploit its prolific secondary metabolism. In *Streptomyces*, the remarkable organization of the chromosome^2,3^, *i.e*. central conserved versus terminal variable regions, questions the recombination mechanisms ensuring genome stability. Early genome comparisons achieved in our lab^4^ showed that variability in the subtelomeres is fueled by the input of exogenous information through horizontal transfer and driven by a very specific recombination pattern along the chromosome, *i.e*. recombination frequencies growing towards the chromosome ends. Moreover, *Streptomyces* are well-known as providers of a wide range of bioactive natural products and constitute an untapped source for new treatments against human microbial infections as well as against cancer. Genes responsible for the biosynthesis of metabolites of interest are usually clustered and frequently located in the terminal regions of the chromosome^33,34^. They are consequently subjected to DNA plasticity. Traces of recombination events are noticeable within the structure and sequence of these gene clusters. Studying genome plasticity can thus give insight into the evolution of the repertoire of secondary metabolism and open the way to its diversification using recombination.

Although HR is known to contribute to genome stability as an error free repair mechanism in all living organisms, our results point out that it is also a main driving force of genome structure plasticity in *Streptomyces*. DSB repair in the terminal regions was most of the time associated with the loss of the broken arm as well as a deletion towards the inner part of the chromosome. In a vast majority of analyzed genomes (15 of 18), chromosome salvage was achieved by chromosomal arm replacement. Two pairs of DNA repeats (*i.e*. HR1-L/HR1-R and HR2-L/HR2-R) present on both arms in opposite orientation allows HR to initiate either a crossing over with the opposite arm of another chromosome present in the mycelium compartment or with the opposite arm of the same chromosome. In the former case, recombination leads to the acquisition of an intact arm, the latter could initiate a break-induced replication (BIR) event^35^. The BIR mechanism is well-known in yeast as a powerful mechanism leading to large segmental duplications of the sub-telomere region. This is also underlying the loss of heterozygosis in mammalian cells^36^.

The stepwise plasticity associated with the loss of *tap/tpg* locus also explains the heterogeneity of the progeny observed over several rounds of sporulation. While phenotypic stability was easily reached when the DSB was induced in the right arm, a strong and persisting heterogeneity imposed to subclone several times the lineages derived after a DSB in the left arm. The successive events required for chromosome stabilization could explain the segregation of different colonial phenotypes. This is reminiscent of the hypervariability described in *S. ambofaciens* and the instability cycle associated with the chromosome fusion^10,37^.

Out of 18 mutant chromosomes analyzed, four demonstrated extensive DNA amplification (Table 1). All occurred in a locus known as AUD90^37^ which was later shown to include a large secondary metabolite gene cluster encoding PKSs involved in the biosynthesis of stambomycins^38,39^. This locus is typified by the presence of numerous repeats corresponding to modular domains of the PKS complex. A close analysis of the boundaries of the amplified unit revealed the presence of short repeated sequences (several hundreds of nucleotides sharing over 90% of identity) that could have been used as substrate for HR. This would support a model proposed by Young and Cullum (1987) in which a replication fork is trapped on the circular amplifiable template formed after recombination between two direct repeats on the chromosome. The implication of HR is also supported by the abolishment of amplification in a *recA* mutant in *S. lividans*^40^. It is interesting to point out that all these amplification cases were associated with an arm replacement event suggesting that the trapped replication fork could have been initiated upstream on the chromosome during a BIR event. Another interesting feature is the circular configuration of the amplified chromosome with large deletion adjacent to the amplification. This implies that the circularization point involves some sequences present in the amplifiable locus. Although no circularization junction could be isolated among the tens of thousands of reads covering this locus in each strain, hindering the identification of the molecular mechanism, the circularization event within this region could be highly frequent because of the high proportion of this amplified DNA in the total genomic DNA. Further, it is not excluded that circularization occurred at several points and by different mechanisms (HR and IR) within the same strain.

Integration of new genetic information can be efficiently achieved through HR between flanking homologous regions. However, HR cannot apply when DNA sequences of donor and receptor are divergent or when incoming DNA does not find any homologous region to recombine with (*e.g*. specific regions of *Streptomyces* chromosome). Hence, illegitimate recombination was reported to favor DNA insertion at recombination points in *E. coli*^28^ and in eukaryotes^41–43^. In *S. ambofaciens*, we identified 5 insertion events at DSB healing sites with size ranging from 30 bp to 18.8 kb. In our experiments, the inserted sequences originated from *S. ambofaciens* genome and were unambiguously identified thanks to their endogenous origin. We speculate that in presence of exogenous DNA (conducted by conjugational processes for instance) NHEJ could play an active role in the integration of new sequences. Interestingly, a correlation between the capacity of acquisition of foreign DNA and the presence of a NHEJ pathway in prokaryotes was underlined by Popa *et al*.^44^. Thus, NHEJ may participate to overcome barriers to horizontal transfer between distantly related organisms.

## Methods

### Bacterial strains

*S. ambofaciens* strains were grown at 30 °C on Soya Flour Mannitol (SFM) plates or in Hickey Tresner (HT) medium for liquid cultures. All *Streptomyces* modified strains derive from our reference strain *S. ambofaciens* ATCC 23877^45^. To isolate lineages following DSB induction, a minimum of three subcloning rounds (up to eight rounds until phenotype homogeneity was reached) was realized on SFM plates supplemented with the appropriate selective antibiotic, then spores were harvested as previously described^46^. The DH5α *E. coli* strain was used as host for native and recombinant BACs and plasmids. The ET12567 non methylating *E. coli* strain containing the mobilizing pUZ8002 plasmid was used as donor for all intergeneric conjugation between *E. coli* and *S. ambofaciens*. *E. coli* strains were grown in Luria-Bertani (LB) medium at 37 °C except for the BW25113 pKD20 thermosensitive strain used for PCR targeting, which was grown at 30 °C. When necessary, antibiotics were added to the medium at a concentration of 50 mg/mL for apramycin, kanamycin and hygromycin, and 25 mg/mL for nalidixic acid and chloramphenicol.

### I-SceI mediated DSB targeting

The coding sequence of yeast endonuclease I-*Sce*I^47^ was optimized for expression in *Streptomyces* and I-*Sce*I gene was cloned under the control of P*tipA* in a conjugative and integrative vector (pDYN6902,^48^. The construct, pDYN6902-*I-*Sce*I*, was transferred from *E. coli* to *S. ambofaciens* by conjugation, where it stably integrates in the chromosome by site-specific recombination at bacteriophage φC31 attachment site (at position 4.11 Mb) (Fig. 1). The basic expression of I-*Sce*I gene in *S. ambofaciens*, *i.e*. in the absence of the inducer thiostrepton, was checked by semi-quantitative RT-PCR. Hence P*tipA* is known to enable a basic expression^49,50^. The I-*Sce*I endonuclease was therefore considered as being expressed in *S. ambofaciens* as soon as the construct was introduced by conjugation. We constructed a target cassette of the endonuclease consisting of the aminoglycoside 3’-phosphotransferase gene (*neo*, conferring the resistance to kanamycin), flanked by two I-*Sce*I sites. The two sites were in a convergent orientation in order to avoid the compatibility of the overhanging DNA extremities produced by the endonuclease. This cassette was named IKI for I-*Sce*I/kan^R^/I-*Sce*I. For the insertion in the central part of the chromosome, the IKI cassette was inserted in the conjugative plasmid pMS82^51^ that integrates by site-specific recombination at the φBT1 attachment site (position 4.94 Mb). This insertion was called IKI-C, C for central region (Fig. 1). To trigger DSBs in the chromosomal arms, the IKI cassette was inserted by homologous recombination into intergenic spaces (positions 0.32 Mb and 7.91 Mb) to generate the insertion sites IKI-L and IKI-R for left and right arm insertion respectively. Briefly, the IKI cassette was inserted by PCR targeting^52^ into a recombinant BAC containing the locus of interest. Transfer of the recombinant BAC by intergeneric conjugation *E. coli/Streptomyces* into *S. ambofaciens* followed by suitable antibiotic selection (kanamycin resistance for the IKI cassette, sensitivity to apramycin for the removal of the BAC) led to the selection for the insertion of the IKI cassette into the targeted intergenic sequence. For all experiments, the IKI cassette insertion was selected before the introduction of the I-*Sce*I expression construct (pDYN6902- I-*Sce*I).

### Molecular analyses

For repair scar or chromosomal structure analyses, Streptomyces genomic DNA was extracted by low binding sulfate salt method as described by Kieser *et al*.^46^. Liquid HT medium was used for growth of mycelium before DNA extraction. To determine the nucleotide sequence of the scars after DSB at IKI-C, the locus was amplified by PCR and the amplicon was gel purified then sequenced by the Sanger method (Beckman Coulter Genomics). For chromosomal structure analyses, a set of PCR amplifications was carried out using primer pairs distributed along the WT chromosome (Fig. 1B) on genomic DNA extracted from strains isolated after DSB induction at IKI-L or IKI-R locus (150 isolates).

### Whole genome sequencing

Genomic DNA was extracted as described above and paired-ends sequencing of short DNA fragments (average size 760 nt) using an Illumina Genome Analyzer was performed for 18 strains (Table S2). Mapping of sequencing reads on genome reference as well as coverage analyses was carried out using CLC Genome Workbench (version 6.5, Qiagen).

### Statistical analyses

Fisher’s exact test was used to test the statistical significance of the different proportions (small samples) throughout the work.

## Electronic supplementary material

Supplementary information
