# Supplementary material for: Genome plasticity is governed by double strand break DNA repair in Streptomyces
Source: Sci Rep. 2018 Mar 27;8:5272. doi: 10.1038/s41598-018-23622-w (PMC5869714; doi:10.1038/s41598-018-23622-w)
Supplement: Supplementary file 1 — Supplementary information [file 41598_2018_23622_MOESM1_ESM.docx]

**Supplementary material**

**Genome plasticity is governed by double strand break DNA repair in *Streptomyces***

Grégory Hoff^1✝^, Claire Bertrand^1^, Emilie Piotrowski^1^, Annabelle Thibessard^1^ and Pierre Leblond^1*^

^1^Université de Lorraine, Institut National de la Recherche Agronomique, Dynamique des Génomes et Adaptation Microbienne (DynAMic), UMR INRA 1128, Nancy, 54000, France

^✝^Present address: Microbial Processes Interactions (MiPI), Gembloux Agro-Bio Tech, Bât. G1 Bio-industries, Passage des Déportés, 25030 Gembloux, Belgium

Figure S1. Colonial phenotypes of strains experiencing DSBs.

Table S1. Spontaneous loss or mutation of I-*Sce*I sites or I-*Sce*I gene

Table S2. Whole genome sequencing data.

Table S3. Recombination events characteristics of the sequenced genomes.

**
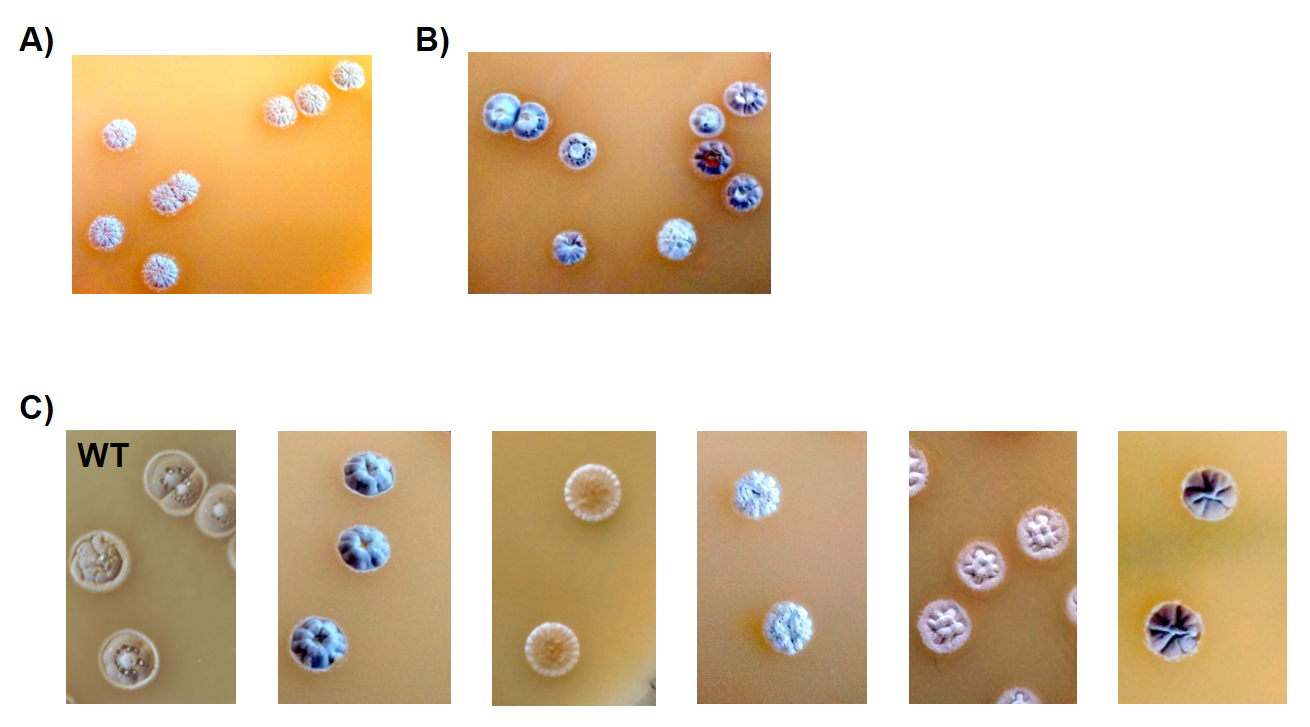
**

**Figure S1. Colonial phenotypes of strains experiencing DSBs.** All the strains were plated on solid mannitol-soya flour medium for 6 days.

**A.** Example of lineage displaying a homogeneous colonial phenotype.

**B.** Example of lineage displaying heterogeneous colonial phenotypes.

**C.** Diversity of colonial phenotype after stabilization (*e.g.* several subclonings after DSB occurrence). WT shows the phenotype of ATCC 23877 before DSB induction and repair.

**Table S1. Spontaneous loss or mutation of I-*Sce*I sites or I-*Sce*I gene**


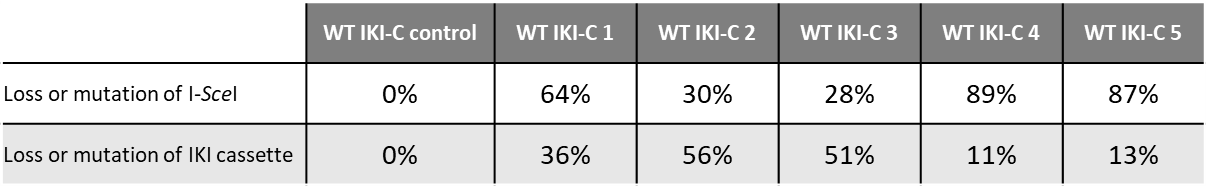


High frequency of spontaneous loss or mutation of the I-*Sce*I sites or I-*Sce*I gene. Five independent WT lineages harbouring at the starting point of the experiments both the IKI (C locus) and I-*Sce*I expression cassettes were surveyed for stability in the absence of the selective pressure (kanamycine and hygromycine for IKI and I-*Sce*I cassettes respectively) after two sporulation rounds. The loss of either or both resistances were the early marker of mutations of the respective loci (100 clones observed for each independent lineage). Further confirmation came from PCR amplification, I-*Sce*I digestion and/or DNA sequencing for the I-*Sce*I cassette, and from gene sequencing for the I-*Sce*I gene. The control consisted of the WT context with the only IKI cassette (10 clones per lineage). In that case, no spontaneous loss of the IKI cassette was observed.

**Table S2. Whole genome sequencing data.**


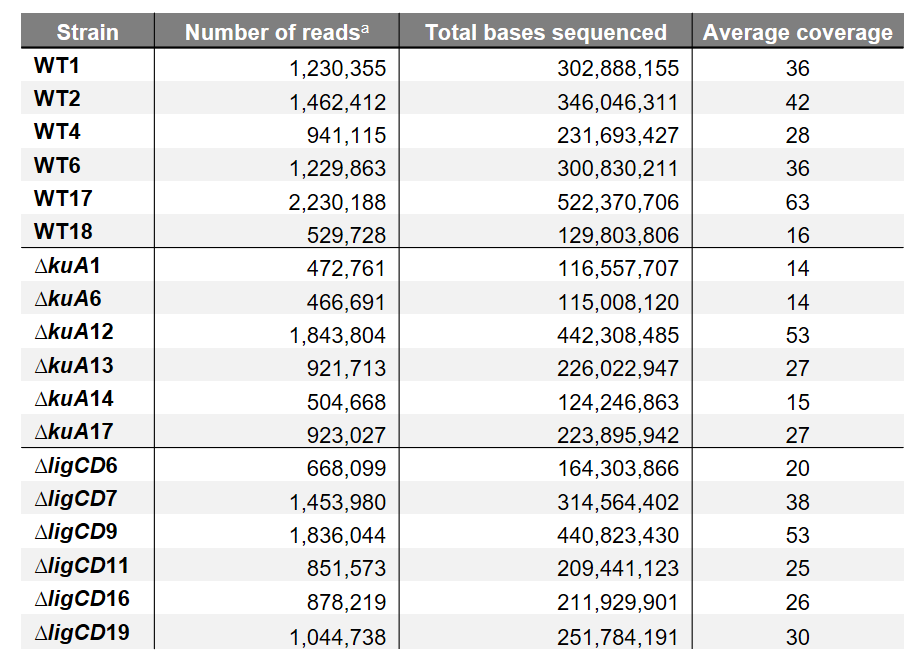
For each strain, a paired-ends genomic library was sequenced using the Illumina technology. The average read length was 240 nt (a). The average coverage corresponds to the ratio of the total of bases sequenced relative to the size of the genome.

**Table S3. Recombination events characteristics of the sequenced genomes.**


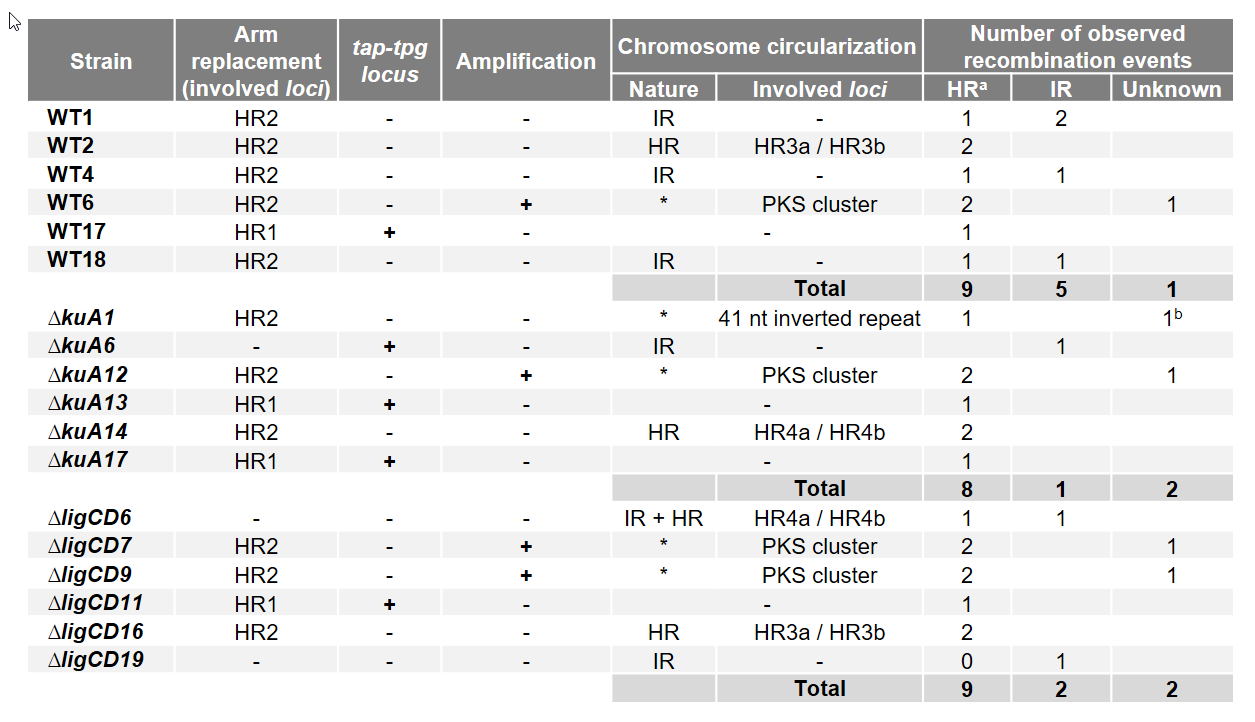
*because of the high number of reads covering the amplified locus, the nature of the event leading to chromosome circularization could not be characterized.

a: the identification of hybrid reads confirmed the HR event hypothesis.

b: the circularization occurred between to inverted repeats of 41 nt, too short to determine the mechanism involved.
